# Long-term survival of cultivated oral mucosal epithelial cells in human cornea: generating cell sheets using an animal product-free culture protocol

**DOI:** 10.1186/s13287-021-02564-7

**Published:** 2021-10-07

**Authors:** David Hui-Kang Ma, Yi-Jen Hsueh, Kevin Sheng-Kai Ma, Yueh-Ju Tsai, Shiang-Fu Huang, Hung-Chi Chen, Chi-Chin Sun, Ming-Tse Kuo, An-Shine Chao, Jui-Yang Lai

**Affiliations:** 1 Limbal Stem Cell Laboratory, Department of Ophthalmology, Chang Gung Memorial Hospital, Linkou, Taiwan; 2 Department of Chinese Medicine, College of Medicine, Chang Gung University, Taoyuan, Taiwan; 3 Center for Tissue Engineering, Chang Gung Memorial Hospital, Linkou, Taiwan; 4 Department of Ophthalmology, Xiamen Chang Gung Hospital, Xiamen, Fujian China; 5 Center for Global Health, Perelman School of Medicine, University of Pennsylvania, Philadelphia, PA USA; 6 Graduate Institute of Biomedical Electronics and Bioinformatics, College of Electrical Engineering and Computer Science, National Taiwan University, Taipei, Taiwan; 7 Department of Dentistry, Chung Shan Medical University and Chung Shan Medical University Hospital, Taichung, Taiwan; 8 Department of Medicine, School of Medicine, College of Medicine, Chang Gung University, Taoyuan, Taiwan; 9 Department of Otolaryngology, Chang Gung Memorial Hospital, Linkou, Taiwan; 10 Graduate Institute of Clinical Medical Science, Chang Gung University, Taoyuan, Taiwan; 11 Department of Ophthalmology, Chang Gung Memorial Hospital, Keelung, Taiwan; 12 Department of Ophthalmology, Kaohsiung Chang Gung Memorial Hospital and Chang Gung University College of Medicine, Kaohsiung, Taiwan; 13 Department of Obstetrics and Gynecology, New Taipei Municipal Tucheng Hospital, Tucheng, New Taipei City Taiwan; 14 Department of Obstetrics and Gynecology, Chang Gung Memorial Hospital, Taoyuan, Taiwan; 15 Graduate Institute of Biomedical Engineering, Chang Gung University, Taoyuan, Taiwan; 16 Department of Materials Engineering, Ming Chi University of Technology, New Taipei City, Taiwan; 17 Research Center for Chinese Herbal Medicine, College of Human Ecology, Chang Gung University of Science and Technology, Taoyuan, Taiwan

**Keywords:** COMET, Collagenase, Oral mucosa, Amniotic membrane, Microsphere

## Abstract

Previously, we reported a collagenase-based, animal product-free protocol for cultivated oral mucosal epithelial cell sheets for transplantation (COMET). Here, we reported the long-term outcomes of first 2 clinical cases. A 27-year-old man suffered from thermal burn, which resulted in symblepharon of lower fornix OD. COMET was performed, and the cornea remained clear with few peripheral NV and no more symblepharon 34 months postoperatively. Another 42-year-old man suffered from severe alkaline burn OD. He underwent COMET, followed by corneal transplantation half a year later. A biopsy taken two years after COMET showed stratified epithelium positive for keratin 4, 13, and 3 in the suprabasal layer. Staining for p63 and p75^NTR^ was both positive in the basal layer. The graft remained clear up to post-OP 4 years. Our study confirmed the long-term survival of the transplanted OMECs, suggesting that collagenase-based spheroidal suspension culture is a promising technique for COMET.

*Trial registration* ClinicalTrials.gov, ClinicalTrials.gov ID: NCT03943797 Registered 9 May 2019-Retrospectively registered, https://clinicaltrials.gov/ct2/show/NCT03943797**.**

**Supplementary Information:**

The online version contains supplementary material available at 10.1186/s13287-021-02564-7.

## Introduction

Rejection is still a major drawback to allograft transplantation. To circumvent allogeneic rejection, previously Nakamura et al. [1] and Nishida et al. [2] reported cultivated oral mucosal epithelial transplantation (COMET) using autologous oral mucosal epithelium as a surrogate for the genuine corneal epithelium to reconstruct the corneal surface after chemical burns and Stevens-Johnson syndrome [3–6]. Stabilization of the corneal surface [7], long-term survival of transplanted oral mucosal epithelial cells (OMECs) [8–11], and improvements in vision have been documented [12].

Despite the success of the technique, earlier protocols invariably used ingredients containing animal products, notably bovine serum and 3T3 feeder cells. Although the risk is small, there is still concern about transmitting zoonoses. Efforts have been made to remove the components derived from animals from the culture medium [13–16].

On the other hand, earlier methods invariably all used dispase II, followed by trypsin/EDTA treatment to isolate OMECs. With this method, many epithelial cells in the tissue were dissociated and became devitalized. Previously, we reported the use of type I collagenase to isolate OMECs from tissue to fabricate an epithelial cell sheet [17]. With this method, the 3T3 feeder cell coculture was no longer needed, and in vitro studies suggested that OMEC sheets generated by collagenase digestion might contain more progenitor cells [17]. A COMET clinical trial using this protocol is currently underway, and initial results suggested that long-term survival of transplanted OMECs is possible.

## Cell culture and transplantation

Human oral mucosal tissue and amniotic membrane (AM) were obtained in accordance with the tenets of the Declaration of Helsinki for research. The clinical trial was approved by the Institutional Review Board of Chang Gung Memorial Hospital, Taiwan’s Food and Drug Administration, and was also registered in ClinicalTrials.gov (ID NCT03943797). Institutional consent form including consent for future publication has been signed by the patients.

The processing of the AM was described in detail previously [17]. Briefly, two layers of 1.5 × 1.5 cm de-epithelialized AM were laid onto a 25-mm culture insert (Corning) and air-dried overnight. An 8 × 8 mm biopsy was taken from the patient’s buccal mucosa under local anesthesia. The tissue was cut into tiny pieces and then added to a 1.5-mL Eppendorf tube containing 1 mL 0.5 mg/mL collagenase A (Roche) in serum-free SHEM.

The tube was kept in an Eppendorf ThermoMixer overnight with rapid shaking at 1,200 rpm and 37 °C. After incubation, the cell suspension was spun down at 4 °C, 3,500 rpm for 5 min, followed by removal of the supernatant. The cell pellet was resuspended in 1.5 mL SHEM containing 5% PLTMax (Merck-Millipore; a human platelet lysate approved for human use) and seeded onto the insert.

Duplicate cultures were made, because one culture will be used for the qualification assay later. Two days later, the medium was replaced with serum-free EpiLife medium (containing 1% Supplement S7; Gibco/Thermo Fisher Scientific). The medium was changed every 3 days. The average cell culture time was 14 days.

Before termination of cell culture, one culture will be chosen for the qualification assays. The procedure of staining the flat mounts was according to previous publications [11, 17]. For characterization, the following antibodies were used: anti-keratin 3 (1: 100; Millipore), anti-keratin 4 (1: 100; Abcam), anti-keratin 13, (1: 100; Abcam), anti-Connexin 43 (1: 50; Millipore), anti-p63 (1: 150; Millipore), and anti-p75^NTR^ (1: 100; Santa Cruz). Staining procedures and photography for the cryosections were described previously [11, 17].

All transplantations were performed under general anesthesia, and the surgical procedure was similar to earlier reports [3, 11, 18]. If the patient has a cataract or residual corneal opacity, cataract extraction or corneal transplantation can be done at least 6 months after COMET.

## Characterization of the cell culture product

Following collagenase treatment, the OMECs became microspheres of variable sizes (Additional file 1: Fig. S1A). Most of the microspheres can attach to the AM, and the epithelial cells then spread out from the microspheres (Additional file 1: Fig. S1B). Individual small cell sheets gradually coalesced to become a confluent sheet by around two weeks (Additional file 1: Fig. S1D–F). The cell sheet exhibited homogenous cytoplasmic staining for keratin 3 (Additional file 1: Fig. S1G) and keratin 13 (Additional file 1: Fig. S1H), but negative staining for keratin 8 (Additional file 1: Fig. S1I; a negative marker for OMECs) [11]. Gap junction protein Connexin 43 was expressed in the intercellular space, but was absent within the microspheres (Additional file 1: Fig. S1J). The cell sheet expressed both p63 (Additional file 1: Fig. S1K) and p75^NTR^ (Additional file 1: Fig. S1L), which were concentrated in the microspheres. Conversely, in these microspheres, the staining for keratin 3 and keratin 13 was negative (not shown).

## Patient 1

**Fig. 1:**
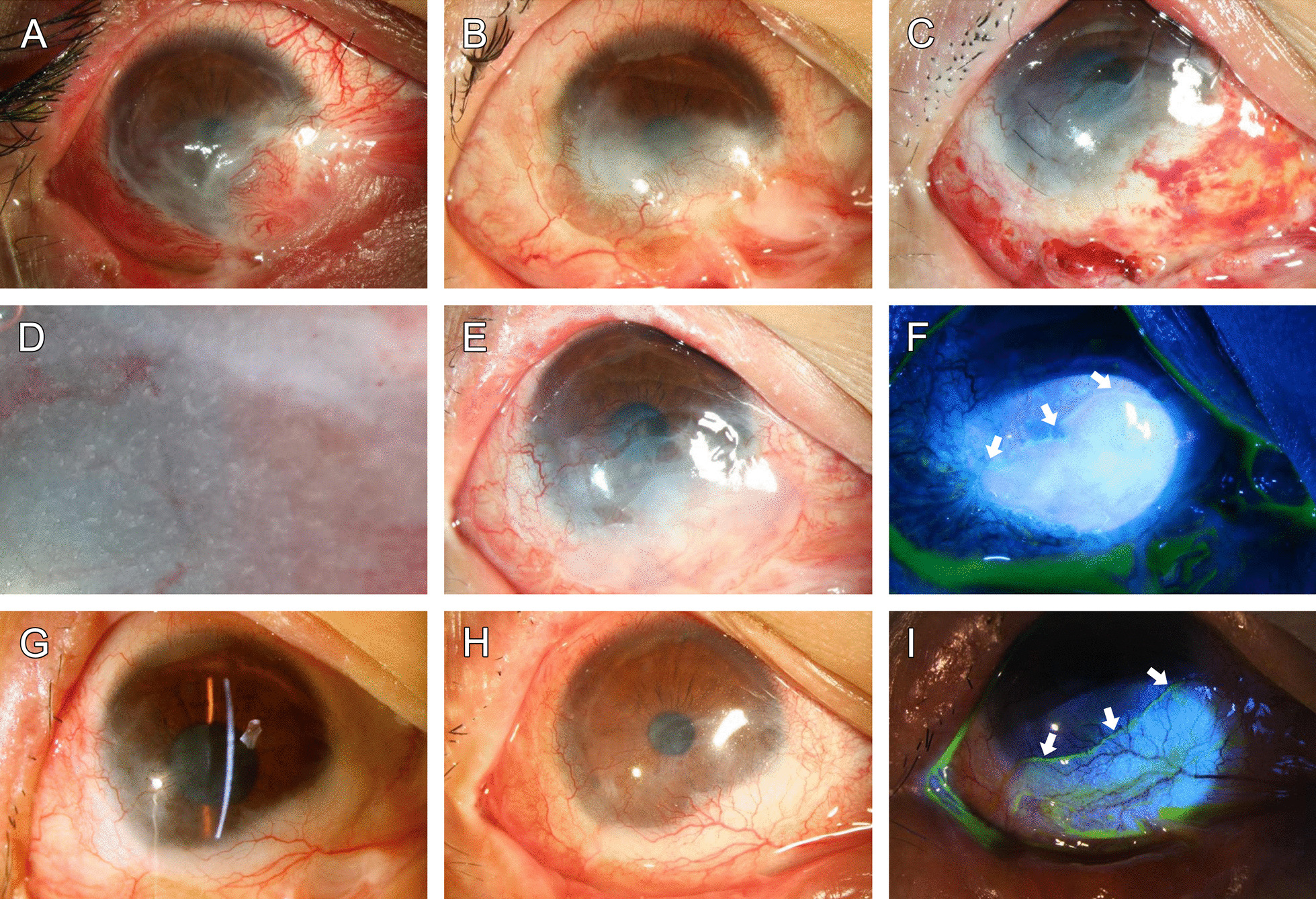
External eye photographs of Patient 1. A 27-year-old man suffered from thermal burn OD caused by molten aluminum. After multiple AM transplantations and tarsoconjunctival grafting, there was still pannus ingrowth and severe symblepharon (**A**). After unsuccessful conjunctivo-limbal autografting, inflammation persisted with fibrovascular invasion and opacification of the lower cornea with symblepharon. His best corrected vision was 20/200 (**B**). One week after COMET, the corneal surface was intact without erosion or defects (**C**). Aggregates of the epithelial microspheres on AM can be seen with a high-magnification slit lamp (**D**). At post-OP 1.5 months, conjunctival inflammation was markedly reduced (E), and fluorescein staining revealed the OMEC sheet covering the lower cornea, limbus, and bulbar conjunctiva (**F**; margin indicated by arrows). One and half a years after COMET, no more symblepharon was seen, and his best corrected vision improved to 20/25 (**G**). At 34 months after transplantation, the cornea remained clear with a few peripheral NV (**H**), and the unique fluorescein staining confirmed the presence of OMECs (**I**; margin indicated by arrows)

A 27-year-old man suffered from thermal burn OD caused by molten aluminum. Despite multiple surgeries, inflammation persisted and fibrovascular tissue reinvaded and resulted in opacification of the lower cornea with symblepharon of the lower fornix, and his best corrected vision OD was 20/300 (Fig. 1A, B).

In early 2017, he received superficial keratectomy and COMET. Postoperatively, the cell sheet remained intact without erosion or defect (Fig. 1C). Aggregates of the epithelial microspheres on AM could be seen by slit lamp (Fig. 1D). The aggregates gradually smoothed out and could no longer be seen 2 weeks postoperatively. At post-op 1.5 months, his conjunctival inflammation was markedly reduced (Fig. 1E, F). One and half a year after COMET, his vision improved to 20/25 by rigid contact lens correction, and no more symblepharon was seen (Fig. 1G). 34 months after transplantation, the cornea remained clear with few peripheral NV, and the unique fluorescein staining of the epithelium over the lower limbus and bulbar conjunctiva indicated the presence of OMECs (Fig. 1H, I).

**Fig. 2:**
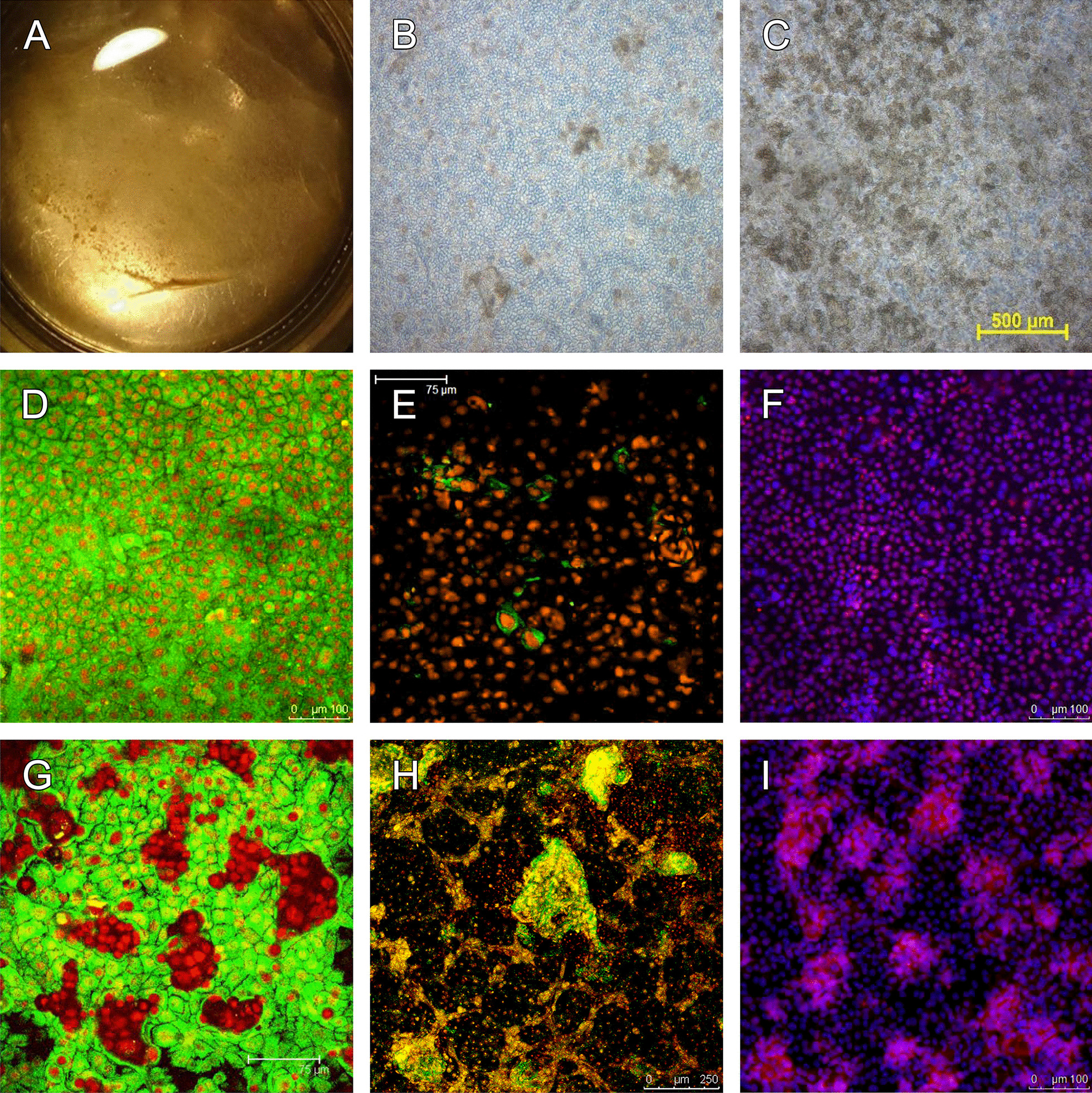
Microscopic and immunostaining photographs of the OMEC sheet from Patient 1. **A** Cell aggregates/microspheres in the cell sheet can be seen under a surgical microscope shortly before transplantation. **B**, **C** Phase contrast microscopic view of the confluent cell sheet in areas with few microspheres (**B**) and with abundant microspheres (**C**). **D**–**F** Immunoconfocal microscopy for keratin 3 (**D**, green; nuclei counterstained with PI), p75^NTR^ (**E**, green), and p63 (**F**, red; nuclei counterstained with DAPI) in areas with few microspheres. **G**–**I** Staining for keratin 3 (**G**, green), p75^NTR^ (**H**, green), and p63 (**I**, red) in areas with abundant microspheres. Note that keratin 3 staining is negative within the microspheres, while signals for p75^NTR^ and p63 are enriched in the structures

In the duplicate culture, cellular aggregates were readily identifiable under a surgical microscope (Fig. 2A). Under a phase-contrast microscope, some areas of the cell sheet were mainly composed of squamous epithelial cells (Fig. 2B), while in other areas, microspheres predominated (Fig. 2C). In the squamous epithelium predominate area, keratin 3 staining was homogenous (Fig. 2D), p75^NTR^ staining was sporadic (Fig. 2E), and p63 staining was discrete and diffuse (Fig. 2F). In the microsphere dominated area, keratin 3 staining was absent within the microspheres (Fig. 2G). In contrast, p75^NTR^ and p63 stainings were concentrated in the cell aggregates. Collectively, these findings suggest the enrichment of progenitor cells within the microspheres/cell aggregates in the OMEC cultures.

## Patient 2

**Fig. 3:**
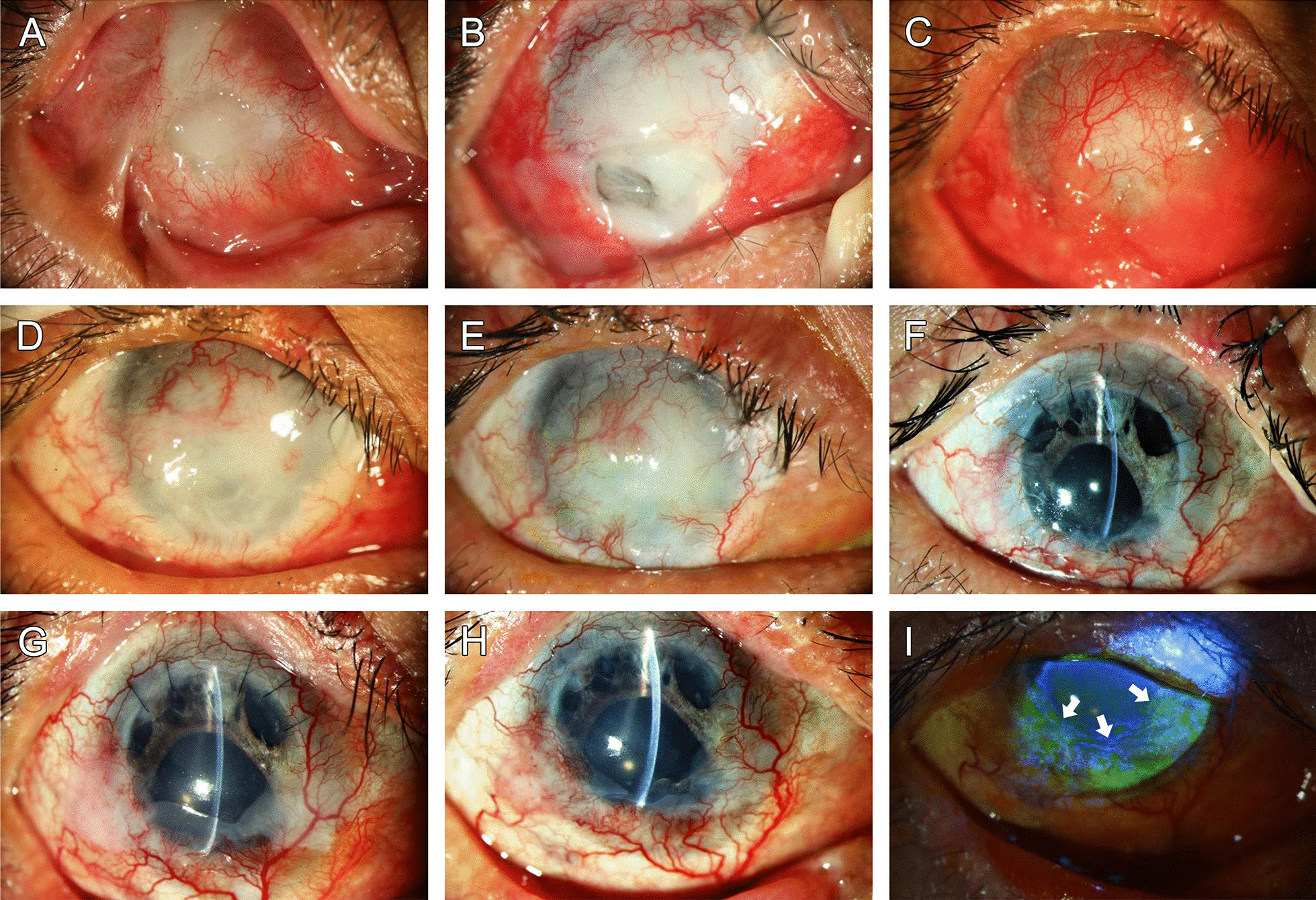
External eye photographs of Patient 2. A 42-year-old man suffered from severe alkaline burn. Extensive pannus ingrowth, entropion and symblepharon were noted (**A**). Conjunctivo-limbal autografting was not successful, and the cornea remained severely inflamed with a persistent epithelial defect, which evolved to become a perforated ulcer (**B**). The ulcer finally healed, but the cornea was covered by dense blood vessels and granulation tissue, and his vision was only light perception (**C**). After COMET, the cornea was well-epithelialized, and there was no single episode of epithelial defect (**D**, postop one month). The cornea was essentially avascular except in the temporo-upper quadrant (**E**, postop 6 months). Six months after COMET, penetrating keratoplasty was performed (**F**, photo taken 3 months postop). Two years after COMET, the graft was clear. **G** The graft remained clear up to post-COMET 4 years, and his best corrected vision remained above 20/120 (**H**). In the lower cornea and limbus, characteristic coarse fluorescein staining confirmed the presence of OMECs (**I**, margin indicated by arrows)

A 42-year-old man suffered from severe alkaline burn (sodium hydroxide) OD. When he was referred to our hospital, entropion and symblepharon were noted (Fig. 3A), the cornea was covered by dense blood vessels and granulation tissue, and his vision was only light perception (3C).

After recruitment for this study, COMET was performed following pannus removal, peritomy, and topical mitomycin C soaking. Postoperatively, the cornea was well-epithelialized, and there was no single episode of epithelial defect (Fig. 3D). To treat remaining corneal opacity, 6 months after COMET, penetrating keratoplasty was performed (Fig. 3F). After keratoplasty, the graft remained clear, and his best corrected visual acuity (BCVA) reached 20/80.

To understand the fate of the transplanted OMECs, 1.5 years after keratoplasty, a biopsy was taken from the lower limbus (Fig. 3G). The graft remained clear up to post-COMET 4 years, and his BCVA remained above 20/120 (Fig. 3H). In the lower cornea and limbus, fluorescein staining confirmed the presence of OMECs, which exhibited a characteristic coarse staining pattern (Fig. 3I) similar to previous findings [3, 11, 18].

**Fig. 4:**
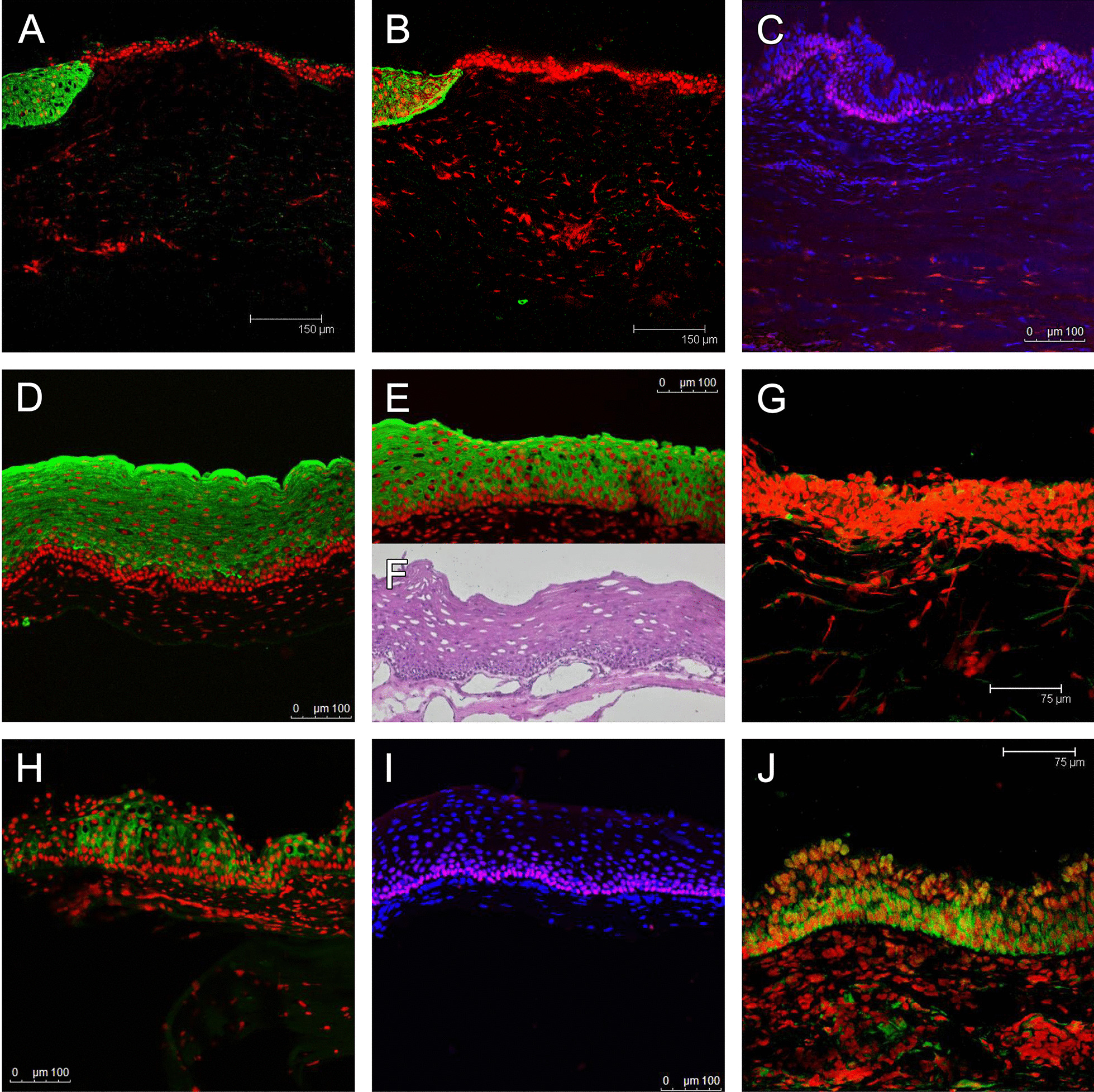
Immunoconfocal microscopy for keratin 12 (**A**, green; nuclei counterstained with PI), 8 (**B**, green), and p63 (**C**, red; nuclei counterstained with DAPI) in a corneal button removed half a year after COMET, and keratin 4 (**D**, green), 13 (**E**, green), 8 (**G**, green; no signal seen), 3 (**H**, green), p63 (**I**, red) and p75^NTR^ (**J**, green) in a limbal biopsy taken 2 years after COMET. Keratin 12 (**A**) and keratin 8 (**B**) were both positive in the upper part (left side) of the corneal button, suggesting the presence of corneal epithelium in this area. While the majority of the specimen was keratin 12 and keratin 8-negative oral mucosal epithelium, p63 signal was universally expressed in the basal epithelium (**C**). Biopsy from the lower limbus 2 years after COMET showed stratified epithelium with over 10 layers of cells in some areas (**F**). The basal epithelial cells were small, compact, and with a high N/C ratio. The epithelium was positive for keratin 4 (**D**), 13 (**E**), and 3 (**H**) in the suprabasal layer but uniformly negative in the basal layer. Negative keratin 8 staining confirmed the origin from the oral mucosa (**G**). Staining for p63 (**I**) and p75^NTR^ (**J**) was both uniformly positive in the basal layer

Immunoconfocal microscopy for keratin 12 (Fig. 4A) and keratin 8 (4B) both showed positive staining in the upper part of the corneal button obtained half a year after COMET, suggesting the presence of corneal epithelium in this area. While the majority of the specimen was keratin 12 and keratin 8-negative oral mucosal epithelium, p63 signal was universally expressed in the basal epithelium (Fig. 4C).

The biopsy taken 2 years after COMET showed stratified epithelium with over 10 layers of cells in some areas (Fig. 4F). The basal epithelial cells were small, compact, and with a high N/C ratio. The epithelium showed positive staining for keratin 4 (Fig. 4D), 13 (Fig. 4E), and 3 (Fig. 4H) in the suprabasal layer but was uniformly negative in the basal layer. Negative keratin 8 staining confirmed the origin from the oral mucosa (Fig. 4G). Staining for p63 (Fig. 4I) and p75^NTR^ (Fig. 4J) was both uniformly positive in the basal layer. This implied that the oral mucosal epithelium rich in progenitor cells persisted in the recipient cornea.

## Discussion

Previously, long-term clinical success after cultivated autologous limbal or oral mucosal epithelial cell transplantation has been reported [7, 8, 19–22]. However, there are only a few studies reporting the histological evidence of the persistence of transplanted cells in the recipient’s cornea [9, 11, 22]. OMECs express keratin 4, 13 but not keratin 8, which distinguishes them from corneal or conjunctival epithelial cells, and therefore, our study provides solid evidence that cultivated epithelial cells can survive for a long time after transplantation.

Previously, Chen et al. reported a new isolation method using collagenase A to isolate human limbal progenitor cells, which maintained a close association with their niche cells [23]. Earlier, we demonstrated that cultivated OMECs isolated by collagenase in the absence of feeder cells exhibited a significantly higher ratio of BrdU label retention, epithelial colony formation, p75^NTR^ and p63-positive cells compared with OMECs isolated by trypsin/EDTA with 3T3 coculture. Pathway analysis pointed to the preferential activation of the integrin beta1/integrin-linked kinase/Wnt signaling pathway [17]. In this article, we further reported the long-term survival of OMECs in recipient corneas after COMET. A very unique feature of the cell sheet generated by collagenase treatment is the presence of microspheres or cell aggregates in the culture, which in some instances were visible even after transplantation (Fig. 1D).

Earlier methods to isolate OMECs from tissue invariably used dispase II, followed by trypsin/EDTA treatment [3–6, 24]. Using this method, we found many epithelial cells after dissociation became devitalized. With collagenase treatment, we found most of the isolated cells transformed into microspheres/cell aggregates. Most of the microspheres could attach to the AM, proliferate and coalesce to become an intact cell sheet. This protocol greatly reduces cell death during isolation, thereby increasing the yield of the cell culture.

Because basement membrane (BM) proteins and stromal (niche) cells are important components of the limbal stem cell niche [25], we hypothesized that following collagenase treatment, the preserved BM proteins and stromal cells reorganize in the OMEC microspheres and function as a surrogate stem cell niche. This is supported by the observation that p75^NTR^ and p63 positive cells are concentrated in the microspheres, with reciprocally negative expression of the keratinocyte differentiation markers keratin 3 and Connexin 43.

Previously, to avoid using 3T3 as feeder cells, human oral [16], dermal [13, 14, 26, 27], and limbal fibroblasts [28] have been used as substitute feeder cells for cultivating OMECs. By using collagenase to isolate OMECs and AM as a scaffold, we found that adequate progenitor cells can be expanded in vitro, and therefore, coculture with feeder cells is no longer necessary. Nevertheless, we found that fibroblast overgrowth can be a problem. Initially, human platelet lysate (PLTMax) was added as an alternative source of serum to facilitate cell attachment, but the medium needs to be changed fairly soon to a serum-free culture medium (EpiLife), which is necessary to control fibroblast overgrowth [17].

In Patient 2, the cornea was initially covered by total vascularization, yet dramatic regression of the vascularization after COMET was observed (Fig. 3D), and the cornea remained clear and avascular long after PKP (Fig. 3H). Keratin12 and 8-positive corneal epithelial cells (Fig. 4A, B) were identified in the corneal button obtained after COMET. This implies that even after severe trauma such as alkaline burn, although grossly the cornea exhibits a clinical picture of total limbal stem cell deficiency with dense vascularization and conjunctivalization, a small number of limbal epithelial stem cells (LESCs) might still hide in deep structures such as limbal crypts [29–31]. Therefore, COMET may not only provide an alternative source of epithelial SCs, it might also ameliorate the limbal microenvironment or provide growth factors to revive the remaining LESCs. Trophic effect following cultivated epithelial transplantation merits further investigation. In addition, although the biopsy taken 2 years after COMET showed considerable p63-positive basal epithelial cells, the immunostaining pattern was not much different from our previous study [11]. Without a reliable control, evidence for the assumption that grafts generated by collagenase isolation might contain more progenitor cells is still lacking; therefore, further investigation is needed.

In summary, we reported successful COMET using a collagenase-based spheroidal suspension culture method. Long-term graft survival is possible with this new protocol. Being free of animal products, with no need of feeder cells, and a higher yield of progenitor cells, this technique shows promise for cultivating various epithelial cells for ocular surface reconstruction.

## Supplementary Information

**Additional file 1: Fig. S1**. Serial phase contrast microscopic photographs (**A**–**F**) and immunoconfocal microscopy (**G**–**L**) for the cell culture. Following collagenase treatment, the OMECs became microspheres of variable sizes, accompanied by a few dissociated single cells (**A**, day 1). Most of the microspheres can attach to the AM, and the epithelial cells then spread out from the microspheres (**B**, day 3). Individual small cell sheets gradually coalesced to become a confluent sheet around two weeks (**C**, day 5; **E**, day 8; **D**, day 11; **F**, day 15). Immunoconfocal microscopy for keratin 3 (**G**, green; nuclei counterstained with PI), 13 (**H**, green), 8 (**I**, green; no signal seen), Connexin 43 (**J**, green), p75^NTR^ (**K**, green), and p63 (**L**, red; nuclei counterstained with DAPI) in a cultivated OMEC sheet. The cell sheet exhibited homogenous cytoplasmic staining for keratin 3 (**G**) and 13 (**H**), but negative staining for keratin 8 (**I**; negative marker for OMECs). Connexin 43 was expressed in the intercellular space but was absent within the microspheres (**J**). The cell sheet expressed both p75^NTR^ (**K**) and p63 (**L**), and the signals were concentrated in the microspheres

## Data Availability

The datasets used and/or analyzed during the current study are available from the corresponding author on reasonable request.
