# Supplementary figures and images for: Long-term survival of cultivated oral mucosal epithelial cells in human cornea: generating cell sheets using an animal product-free culture protocol
Source: Stem Cell Res Ther. 2021 Oct 7;12:524. doi: 10.1186/s13287-021-02564-7 (PMC8496076; doi:10.1186/s13287-021-02564-7)

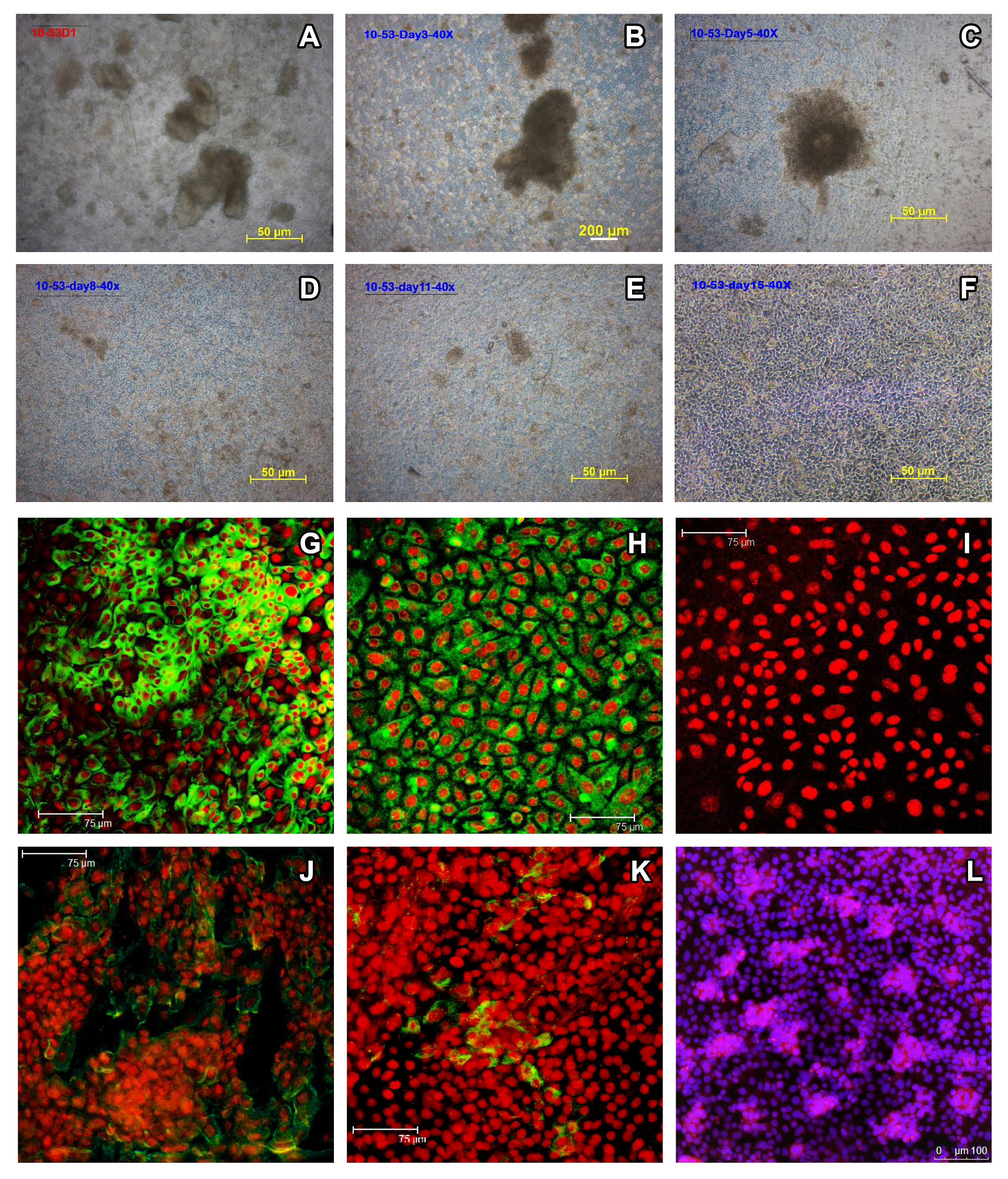

Supplement: Supplementary file 1 — Additional file 1: Fig. S1. Serial phase contrast microscopic photographs (A–F) and immunoconfocal microscopy (G–L) for the cell culture. Following collagenase treatment, the OMECs became microspheres of variable sizes, accompanied by a few dissociated single cells (A, day 1). Most of the microspheres can attach to the AM, and the epithelial cells then spread out from the microspheres (B, day 3). Individual small cell sheets gradually coalesced to become a confluent sheet around two weeks (C, day 5; E, day 8; D, day 11; F, day 15). Immunoconfocal microscopy for keratin 3 (G, green; nuclei counterstained with PI), 13 (H, green), 8 (I, green; no signal seen), Connexin 43 (J, green), p75NTR (K, green), and p63 (L, red; nuclei counterstained with DAPI) in a cultivated OMEC sheet. The cell sheet exhibited homogenous cytoplasmic staining for keratin 3 (G) and 13 (H), but negative staining for keratin 8 (I; negative marker for OMECs). Connexin 43 was expressed in the intercellular space but was absent within the microspheres (J). The cell sheet expressed both p75NTR (K) and p63 (L), and the signals were concentrated in the microspheres [file 13287_2021_2564_MOESM1_ESM.tiff]
